# Toxicity and Molecular Identification of Green Toadfish *Lagocephalus lunaris* Collected from Kyushu Coast, Japan

**DOI:** 10.1155/2011/801285

**Published:** 2011-10-19

**Authors:** Yuji Nagashima, Takuya Matsumoto, Keisuke Kadoyama, Shoichiro Ishizaki, Makoto Terayama

**Affiliations:** ^1^Department of Food Science and Technology, Tokyo University of Marine Science and Technology, Konan 4-5-7, Minato, Tokyo 108-8477, Japan; ^2^Aquaculture and Food Technology Division, Miyazaki Prefectural Fisheries Experimental Station, Aoshima 6-13-6, Miyazaki 889-2162, Japan

## Abstract

Green toadfish *Lagocephalus lunaris* inhabits tropical and subtropical seas and contains high tetrodotoxin (TTX) levels in the muscle as well as liver and gonad. In 2008 to 2009, food poisoning due to ingesting *L. lunais* occurred in Western Japan. Five specimens of green toadfish caught in Kyushu coast, Japan, were analyzed for toxicity, toxins, and species identification. All five specimens were toxic by bioassay. Comparing the maximum toxicity in tissues, ovary contained the most toxin (1810 mouse unit [MU]/g), followed by liver (341 MU/g), muscle (135 MU/g), skin (79 MU/g), and intestine (72 MU/g). Liquid chromatography/mass spectrometry analysis revealed that TTX was the major toxin. Nucleotide sequence analysis of the 16S rRNA gene fragment of muscle mitochondrial DNA indicated that partial sequences of PCR products of four specimens were identical with that of *L. lunaris*. The sequence of one specimen was indistinguishable from that of the brown-backed toadfish *Lagocephalus wheeleri*, a nontoxic species.

## 1. Introduction

Food poisoning incidents due to ingestion of toxic green toadfish *Lagocephalus lunaris* consecutively occurred in Western Japan in 2008 to 2009. As shown in Table 1, a total of 5 incidents including 11 victims and no death were documented. The patients exhibited symptoms similar to tetrodotoxin (TTX) poisoning, such as paralysis, nausea, vomiting, and ataxia. It is well known that puffer fish belonging to the family Tetraodontidae has a high level of TTX in liver and ovary. There are as many as 50 species of Tetraodontidae in the coasts of Japan. Among them green toadfish *L. lunaris* is a notorious species, because it contains high toxin level in even muscle and has caused severe food poisoning [1–3]. The first case was reported in 1959 when five persons ate a few pieces of the fried flesh of green toadfish* L. lunaris* caught in the Vietnam Sea and developed typical signs and symptoms of TTX poisoning [4, 5]. 

Green toadfish *L. lunaris* usually distributes in tropical and subtropical seas including the East China Sea, the South China Sea, and the Indian Ocean but rarely appears in Japanese off coasts, temperate waters [6–12]. Therefore, less attention has been paid to* L. lunaris* in Japan. However, it is notable that the puffer fish poisoning incidents in 2008 and 2009 resulted from *L. lunaris *caught in coasts of Japan, and the patients misidentified it as brown-backed toadfish *Lagocephalus wheeleri.* It is difficult to distinguish a toxic species *L. lunaris* from a nontoxic species *L. wheeleri *that is allowed to eat in Japan, because the two species are closely similar to each other in external morphology [13] and often caught together [12]. In this study, we analyzed toxicity and toxins of *L. lunaris* collected from the Kyushu coast, Japan, and identified the puffer fish by PCR amplification method from the view point of food hygiene. 

## 2. Materials and Methods

### 2.1. Materials

**Figure 1:**
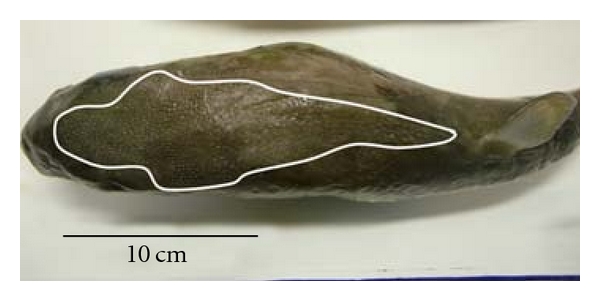
Dorsal side of green toadfish specimen. The spines extend to the base of the dorsal fin.

Puffer fish specimens were caught by longline fishing in the Kyushu coast, Japan, on March 2001, November 2008, and February 2009. They were immediately frozen, transported to the Laboratory of Tokyo University of Marine Science and Technology, and stored at −20°C until use. The puffer fish extending spines to the base of the dorsal fin were recognized as green toadfish *L. lunaris* according to the morphological identification [13]. A typical example of *L. lunaris* is shown in Figure 1.

### 2.2. Assay of Toxicity

After partially thawed, each specimen was dissected into five tissues such as muscle, skin, liver, intestine, and gonad (ovary or testis). The tissue samples were ground in a mortar with a pestle and homogenized with 0.1% acetic acid. TTX was extracted by heating in a boiling water bath for 10 min according to the official guidance of the Japan Food Hygiene Association [14]. The toxicity of each sample was measured by bioassay using four-week-old male ddY strain mice weighing 20 g, following the above official guideline [14]. The toxicity in the mouse bioassay was expressed as mouse unit (MU) where one MU is defined as the amount of toxin that kills a mouse in 30 min after intraperitoneal injection. All the animal experiments were performed in compliance with the fundamental guidelines for proper conduct of animal experiment and related activities in academic research institutions under the jurisdiction of the Ministry of Education, Culture, Sports, Science and Technology and approved by the animal experiment committee in the Tokyo University of Marine Science and Technology. 

### 2.3. Toxin Analysis

Aliquots of the tissue extracts from sample no. 2 in Table 2 were ultrafiltered through a Vivaspin 500 (MWCO 5000, VivaScience AG, Hannover, Germany). The resulting filtrate was subjected to liquid chromatography/electron spray ionization-mass spectrometry (LC/ESI-MS) and analyzed for their toxin profiles as previously reported [15]. Briefly, LC/ESI-MS was performed on an alliance Zspray MS 4000 LC/ESI-MS system (Waters, Milford, Mass, USA). The analytical column was a Develosil C30-UG-5 (1.5 × 250 mm, Nomura chemical, Seto, Japan) and maintained at 25°C. The mobile phase was 20 mM heptafluorobutyric acid in 10 mM ammonium formate (pH 4.0) containing 1% acetonitrile and eluted at a flow rate of 0.10 mL/min. The eluate was induced into the ion source block of ESI-MS detector and ionized by the positive ion mode with desolvation temperature at 350°C, ion source block temperature at 100°C, and cone voltage at 45 kV. 

### 2.4. DNA Extraction and PCR Amplification of Mitochondrial 16S rRNA Gene Fragment

Total cellular DNA was extracted from muscle of each specimen with a DNeasy Blood & Tissue kit (Quiagen K.K., Tokyo, Japan) by manufacturer's instructions. In brief, 25 mg aliquots of ordinary muscle were mixed with 180 *μ*L Buffer ATL and 40 *μ*L proteinase K solution, incubated at 55°C for 1 h, and centrifuged at 20,000 ×g for 15 min. The resulting supernatants were treated with 4 *μ*L RNase A (100 mg/mL) for 2 min, followed by adding 200 *μ*L Buffer AL to incubate at 70°C for 10 min and then adding 200 *μ*L ethanol. DNA was purified with a DNeasy Mini Spin column. The preparations were subjected to the column, washed with each 500 *μ*L Buffer AW1 and Buffer AW2, successively, and eluted with 200 *μ*L AE Buffer. 

A partial region (about 615 bp) of the mitochondrial 16S rRNA gene was amplified by the conventional PCR using universal primers (16SarL, 5′-CGCCTGTTTATCAAAAACAT-3′ and 16SbrH, 5′-CCGGTCGAAACTCAGATCACGT-3′) [16]. PCR was performed in 50 *μ*L total volume of reaction buffer containing 4 *μ*L 2.5 mM dNTPs, 1.5 *μ*L 20 *μ*M of each primer, 0.4 *μ*L EXTaq DNA polymerase, 5 *μ*L 5 × EXBuffer, and 5 *μ*L extracted template DNA (1 *μ*g). PCR was carried out with a thermal cycler PC-801 (Astec, Fukuoka, Japan). Amplifying conditions were 98°C for 30 s in denaturing, 53°C for 30 s in annealing, and 70°C for 60 s in extension for 30 cycles. The PCR products were analyzed by electrophoresis in a 2% agarose gel containing SYBR Safe DNA Gel Stain (Invitrogen, Carlsbad, Calif, USA) and observed with a luminescent image analyzer (LAS-4000 mini, Fujifilm Cooperation, Tokyo, Japan).

### 2.5. DNA Sequencing

After amplification, the PCR products were used as a template for direct sequencing. DNA was sequenced with ABI PRISM 3130 genetic analyzer (Applied Biosystems, Foster, Calif, USA). To identify the puffer fish species, the sequences were searched against DNASIS Taxon V3.0 for Fugu (Hitachi Solutions Ltd., Tokyo, Japan) and the original database of puffer fish mitochondrial sequences in our laboratory of Tokyo University of Marine Science and Technology.

## 3. Results and Discussion

Toxicity of the green toadfish is shown in Table 2. All five individuals showed toxicity by bioassay, although there was marked individual variation in toxicity. Comparing the maximum toxicity in the organs, the ovary was the highest at 1810 MU/g, followed by liver (341 MU/g), muscle (135 MU/g), skin (79 MU/g), and intestine (72 MU/g). The toxicity of puffer fish is classified into four levels, based on the estimated minimum lethal dose (10,000 MU) of TTX in human; extremely strongly toxic (more than 1000 MU/g), strongly toxic (100–999 MU/g), weakly toxic (10–99 MU/g), and nontoxic (below 10 MU/g). Accordingly, the toxicity level of ovary was regarded as extremely strongly toxic, muscle and liver as strongly toxic, and skin and intestine as weakly toxic. It should be noted that two muscle samples (nos. 2 and 4) of five tested showed the strongly toxic level exceeding 100 MU/g, comparing to those in the East China Sea (261 MU/g), Taiwan (140 MU/g), and Thailand (243 MU/g) [8, 10, 11]. These results indicate that the green toadfish obtained in the Kyushu coast was also enough toxic to cause puffer fish poisoning by ingesting muscle and that it is hazardous for human consumption as well as that in tropical and subtropical waters. Another concern is whether the toxic green toadfish accidentally migrates to coastal regions of Japan or inhabits them throughout the year.

**Figure 2:**
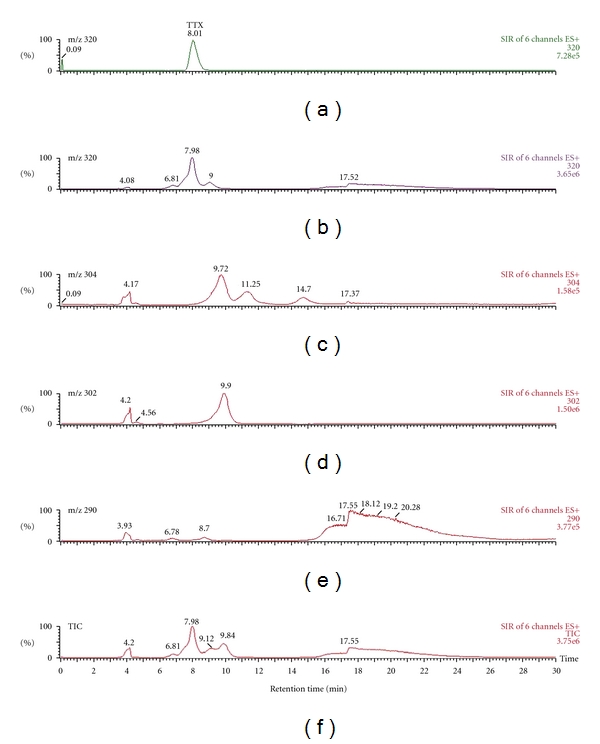
LC/ESI-MS of TTX standard (top) and the muscle extract of sample no. 2.

Figure 2 illustrates LC/ESI-MS of the muscle extract of sample no. 2. The mass chromatograms were scanned at m/z 320 for TTX (C_11_H_17_O_8_N_3_, 319.27 Da), m/z 304 for deoxyTTX (C_11_H_17_O_7_N_3_, 303.27 Da), m/z 302 for anhydroTTX (C_11_H_15_O_7_N_3_, 301.26 Da), and m/z 290 for norTTX (C_10_H_15_O_7_N_3_, 289.25 Da). In the selected ion mass chromatogram at m/z 320, the peak at a retention time of 7.98 min was consistent with that of TTX standard at a retention time of 8.01 min (Figure 2, the top). The peaks at a retention time of 9.72 min at m/z 304 and that of 9.90 min at m/z 302 were estimated to be deoxyTTX and anhydroTTX; respectively, although they were not identified in detail because of lack of the standard of TTX analogues. Total ion current mass chromatogram demonstrated that TTX was a major toxic principle in the muscle extract (Figure 2, the bottom). The liver extract of sample no. 2 showed the same toxin profile as that of the muscle extract (data not shown). Toxin amounts of the sample extracts estimated by LC-ESI/MS were well related to toxicity scores assessed by bioassay. Brillantes et al. detected TTX along with 4-epi-TTX and 4,9-anhydroTTX in muscle and liver extracts from *L. lunaris* in Thailand by HPLC analysis [11]. Moreover, Ngy et al. determined TTX as the major toxin and anhydroTTX as the minor in the Cambodian *L. lunaris* by LC/ESI-MS [12]. In addition, they reported no detectable paralytic shellfish toxins in *L. lunaris* specimens from Thailand and Cambodia. It is likely that the green toadfish *L. lunaris* preferably accumulates TTX and its analogues. 

**Figure 3:**
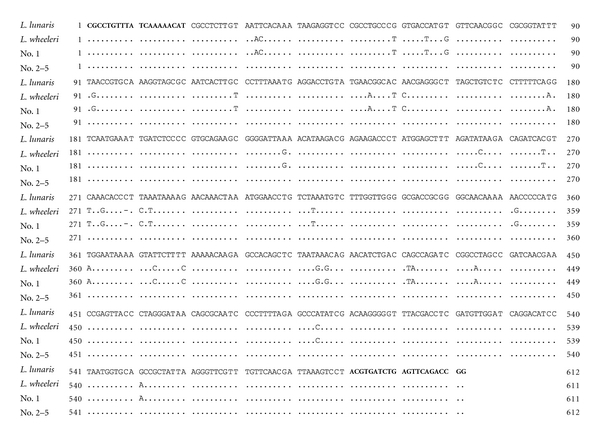
Aligned DNA sequences of the amplified 16S rRNA region of mitochondrial DNA from puffer fish. The positions of the primers 16SarL and 16SbrH used for PCR amplification and sequencing are indicated in bold typeface. A dot (.) indicates identity with *L. lunaris* sequence. A gap introduced into the sequences to optimize the alignment is represented by a dash (-).

 Species identification of the puffer fish specimens was carried out by a direct DNA sequencing analysis according to the method of Ishizaki et al. [17]. Total DNA was isolated from the muscle of green toadfish specimens used for toxicity test and subjected to mitochondrial 16S rRNA gene specific PCR using 16SarL and 16SbrH primers.  Figure 3 shows aligned DNA sequences of the amplified partial 16S rRNA region from the samples, along with the authentic DNA sequences of green toadfish *L. lunaris *and brown-backed toadfish *L. wheeleri*. The PCR products of *L. lunaris *and *L. wheeleri* had a length of 612 bp and 611 bp, respectively. The latter deleted a nucleotide at a site of 279th in *L. lunaris *sequence and had thirty substitutions in nucleotide sequences of *L. lunaris*. 

The partial sequences of the PCR products from four samples (nos. 2–5) were identical with that of *L. lunaris*. However, the sequence of sample no. 1 with no toxicity except for ovary did not agree with that of *L. lunaris*, but* L. wheeleri*, nontoxic species, indicating that sample no. 1 was not regarded as green toadfish by molecular identification, despite broad extension of spines in the back. It is possible that sample no. 1 could be a hybrid of *L. wheeleri* and other, since natural hybrids within the genus of puffer fish have been frequently found [18, 19]. Further study is needed to identify the species in detail by microsatellite DNA analysis and by morphological characterization.

Food poisoning from a dried dressed fish fillet and an adulterated dried mullet roe occurred in Taiwan. Hwang and coworkers identified the causative fish as* L. lunaris* by nucleotide sequence analysis and PCR-restriction fragment length polymorphism using the sequence of 376-nucleotide region in cytochrome *b* gene of mitochondrial DNA [1, 3]. In this study, we analyzed a 16S rRNA gene fragment of mitochondrial DNA and confirmed that 16S rRNA markers are useful and applicable to identify puffer fish species.

## Figures and Tables

**Table 1:** Recent food poisoning incidents due to green toadfish *Lagocephalus lunaris* in Japan.

Date	Place	Number of ingestion	Number of patient
August 18, 2008	Miyazaki Pref.	6	3
August 18, 2008	Kochi Pref.	5	3
August 19, 2008	Kochi Pref.	1	1
October 10, 2008	Kagoshima Pref.	2	2
October 5, 2009	Kagoshima Pref.	2	2

Total		16	11

**Table 2:** Toxicity of green toadfish *Lagocephalus lunaris* caught in the Kyushu coast, Japan.

Sample no.	Date of sampling	Toxicity (mouse unit/g)					
		Muscle	Skin	Liver	Intestine	Ovary	Testis
1	February, 2009	<5	<5	<5	<5	29	
2	November, 2008	109	79	341	72	1810	
3	November, 2008	15	26	143	7	302	
4	March, 2001	135	41	110	62	362	
5	March, 2001	8.2	15	35	14		<5
